# Constitutive ablation of caspase-6 reduces the inflammatory response and behavioural changes caused by peripheral pro-inflammatory stimuli

**DOI:** 10.1038/s41420-018-0043-8

**Published:** 2018-03-12

**Authors:** Safia Ladha, Xiaofan Qiu, Lorenzo Casal, Nicholas S. Caron, Dagmar E. Ehrnhoefer, Michael R. Hayden

**Affiliations:** 1 Centre for Molecular Medicine and Therapeutics (CMMT), CFRI, Department of Medical Genetics, University of British Columbia, 950 West 28th Avenue, Vancouver, BC V5Z 4H4 Canada; 2 Present Address: BioMed X Innovation Center, Im Neuenheimer Feld 515, 69120 Heidelberg, Germany

## Abstract

Traditionally, the family of caspases has been subcategorised according to their respective main roles in mediating apoptosis or inflammation. However, recent studies have revealed that caspases participate in diverse cellular functions beyond their canonical roles. Caspase-6 (C6) is one such protease known for its role as a pro-apoptotic executioner caspase and its aberrant activity in several neurodegenerative diseases. In addition to apoptosis, C6 has been shown to regulate B-cell activation and differentiation in plasma cells as well as macrophage activation. Furthermore, C6 has recently been postulated to play a role in mediating the inflammatory response through the production of TNF-α. In this study we further examine the role of C6 in mediating the inflammatory response and its contribution to the manifestation of behavioural abnormalities in mice. We find that C6 is a positive regulator of TNF-α transcription in macrophages and that ablation of C6 reduces lipopolysaccharide (LPS)-induced TNF-α levels in plasma. Furthermore, loss of C6 attenuates LPS-induced behavioural changes in mice and protects neurons from cytokine-mediated toxicity. These data further support the involvement of C6 in the inflammatory response and point to a previously unknown role for C6 in the pathophysiology of depression.

## Introduction

Caspases are a conserved family of endoproteases that play a central role in regulating cell death and inflammation. Caspase-6 (C6) is traditionally classified as an effector or executioner caspase, based on its pro-apoptotic activity in cleaving other caspases as well as structural proteins resulting in the demise of the nucleus and cell^1–5^. Importantly, C6 has been demonstrated as a key mediator of several non-apoptotic processes, among which is its role in facilitating axonal pruning during development^6–9^. Furthermore, C6 is also responsible for regulating B-cell activation and differentiation into plasma cells^10^, and is also involved in innate immunity in alveolar macrophages, where it cleaves IRAK-M, leading to the release of TNF-α upon an inflammatory challenge^11^.

Inflammation is a complex, indispensable cellular process involved in tissue injury, repair and the maintenance of homeostasis but its persistent activation contributes to a large number of human diseases, including psychiatric and neurodegenerative diseases. Inflammasome assembly is a key molecular event in the inflammatory response and leads to the activation of caspase-1 (C1), which subsequently results in the cleavage and release of numerous cytokines^12^. However, recent evidence suggests C1 may not be the sole caspase involved in the execution of pathways leading to inflammation. Recently, a role for C6 in mediating neuroinflammation has been postulated, whereby C6 can be activated by the neuronal NRLP1 inflammasome through a C1-dependent pathway leading to the release of IL-1β along with C6-mediated axonal degeneration^13^. Further, the overexpression of C6 in the mouse hippocampus increases astrocytic and microglial activation, canonical signs of inflammation in the CNS^14^. In the spinal cord, C6 activity is associated with microglial TNF-α release and hypersensitivity to inflammatory pain^15^. Neuroinflammation is a shared symptom between a number of neurological conditions, from acute injuries such as stroke or traumatic brain injury to psychiatric and neurodegenerative disease^16^. The initial inflammatory stimulus can differ widely and range from infection to ‘sterile’ injuries caused by damage-associated molecular patterns (DAMPs) to catecholamine-mediated monocyte and microglial activation induced by psychological stress^17–20^.

Inflammatory signals generated in the periphery can be transmitted to the brain through several pathways, such as the movement of peripheral pro-inflammatory cytokines across the blood−brain barrier (BBB) or the binding of cytokines to peripheral nerve fibres that eventually result in the production of central cytokine signals^21^. More recently, it has been observed that peripheral immune cells such as monocytes are able to directly invade the CNS following peripheral inflammation, causing neuroinflammation and neurological phenotypes^22^.

In this study, we explore further the role of C6 in mediating inflammation in cells of the peripheral immune system as well as the CNS. We find that the absence of C6 alters TNF-α expression and release following pro-inflammatory stimuli in peripheral tissues and provide evidence suggesting that *C6*^*−/−*^ mice are protected from endotoxin-induced behavioural despair, a phenotype that depends on the secretion of pro-inflammatory cytokines^23^. In the CNS, the loss of C6 renders neurons more resistant to pro-inflammatory and excitotoxic stimuli, thus indirectly reducing neuronal damage-induced microglial activation.

## Results

### Peritoneal macrophages from C6^−/−^ mice display altered TNF-α gene expression

Recently, a number of publications have hinted towards a role for C6 in inflammatory pathways both in the CNS and in peripheral tissues^11,13–15^. In some of these studies, C6 has been identified as a downstream effector that is activated by the pro-inflammatory C1^13,14^.

**Fig. 1:**
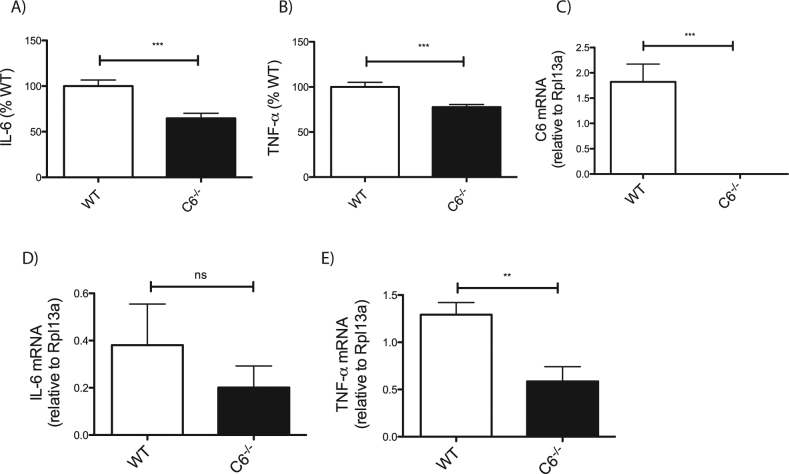
*C6*^−^^*/−*^ mice display reduced plasma levels of TNF-α and decreased *TNF-α* expression. ELISA quantification of plasma cytokine levels in WT and *C6*^*−/−*^ mice at baseline reveal significant lower levels of IL-6 (**a**, *p* = 0.0003, *N* = 18) and TNF-α (**b**, *p* = 0.0006, *N* = 18). Peritoneal macrophages were isolated from WT and *C6*^*−/−*^ mice following pre-treatment with Brewer’s thioglycollate and analysed by quantitative real-time PCR. Gene expression analysis demonstrates that *C6* is expressed in peritoneal macrophages (**c**, *p* = 0.0009, *N* = 5). While *IL-6* gene transcription does not differ between WT and *C6*^−^^*/−*^ mice (**d**, *p* = 0.3881, *N* = 5), *C6*^*−/−*^ macrophages show a significant reduction in *TNF-α* expression (**e**, *p* = 0.0095, *N* = 5). Quantification of target gene is expressed relative to endogenous expression of *Rpl13a*. Statistical significance was determined using a Student’s *t* test. *p < 0.05, **p < 0.01, ***p < 0.001

While the contribution of C1 to the release of pro-inflammatory cytokines is well established, we wanted to determine whether C6 activity directly contributes to inflammatory phenotypes. Given that IL-6 and TNF-α are reported to be two of the most reliable biomarkers of inflammation in neurological disease^21^, we asked whether the loss of C6 would alter the production of these cytokines. Analysing plasma levels of circulating cytokines at baseline in 2–3-month-old WT and *C6*^*−/−*^ mice, we found a significant reduction of both IL-6 and TNF-α in the absence of C6 (Fig. 1a, b).

Since macrophages are the primary immune cell responsible for the production and release of cytokines in the periphery^24^, we next decided to assess cytokine gene expression in peritoneal macrophages. We used peritoneal macrophages as a source of primary cells, as they are readily available and exhibit a mature macrophage phenotype^25^. WT and *C6*^*−/−*^ mice were treated with 3% Brewer’s thioglycollate to recruit and increase the yield of macrophages to the peritoneal cavity^26^, followed by isolation of the cells through lavage.

While C6 expression has been confirmed in alveolar macrophages^11^, its expression in peritoneal macrophages has not been reported. We therefore began with gene expression analyses to confirm that *C6* is indeed expressed in thioglycollate-elicited WT peritoneal macrophages and absent in cells isolated from *C6*^*−/−*^ mice (Fig. 1c). We then quantified the expression of *IL-6* and *TNF-α* genes in these peritoneal macrophages and found no significant differences in the expression of *IL-6* between WT and *C6*^*−/−*^ mice (Fig. 1d). However, peritoneal macrophages derived from *C6*^−/−^ mice had significantly reduced expression of *TNF-α* compared to those from WT mice (Fig. 1e). These data may reflect differential mechanisms of transcriptional regulation and suggest that the loss of C6 results in a reduction in transcriptional activity of the *TNF-α* gene, which in turn would reduce TNF-α protein secretion and its plasma levels at baseline.

### C6^−^^/−^ mice have altered TNF-α plasma levels following treatment with LPS

**Fig. 2:**
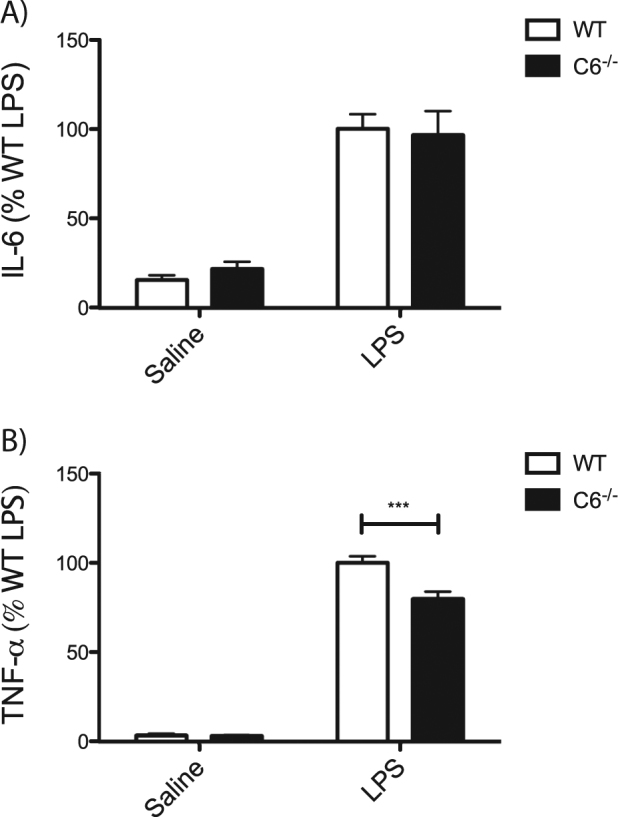
*C6*^−^^*/−*^ mice have reduced TNF-α release following treatment with LPS. ELISA quantification of plasma cytokine levels in WT and *C6*^*−/−*^ mice treated with saline or LPS reveal no differences in IL-6 levels (**a**, genotype *p* = 0.8710; treatment *p* < 0.0001; interaction *p* = 0.5622; *N* = 9) but reduced TNF-α production (**b**, genotype *p* = 0.0009; treatment *p* < 0.0001; interaction *p* = 0.0013; *N* = 9). Statistical significance was determined using two-way ANOVA with post-hoc Bonferroni tests. *p < 0.05, **p < 0.01, ***p < 0.001

We next wanted to assess the impact of inflammation on cytokine production in *C6*^−^^*/−*^ mice, to determine whether baseline differences in TNF-α would be maintained after an acute pro-inflammatory trigger. A commonly used strategy to induce the inflammatory response in rodents is the administration of lipopolysaccharide (LPS), an endotoxin isolated from gram-negative bacteria that activates the immune system^27,28^. WT and *C6*^*−/−*^ mice were thus treated with LPS and plasma was analysed for the concentration of cytokines. Following LPS treatment, both WT and *C6*^*−/−*^ mice demonstrate similarly elevated plasma levels of circulating IL-6 (Fig. 2a). However, the increase in TNF-α is less pronounced in *C6*^−^^*/−*^ compared to WT mice (Fig. 2b), suggesting that the loss of C6 attenuates the increased production of this cytokine during the inflammatory response. This is consistent with our finding that TNF-α expression is specifically reduced in *C6*^*−/−*^ macrophages, as this cell type is expected to contribute significantly to the increase in plasma cytokine levels after LPS treatment^29^.

### C6^−^^/−^ mice are protected against LPS-induced depressive-like behaviour

As C6 is implicated in the production of TNF-α, and the expression of TNF-α along with other cytokines contributes to the manifestation of depression^30–33^, we next asked whether *C6*^*−/−*^ mice would be susceptible to inflammation-driven depressive-like behaviour. In concert with the induction of the inflammatory response, treatment of rodents with LPS also induces depressive-like symptoms such as behavioural despair and anhedonia^23,34–37^.

**Fig. 3:**
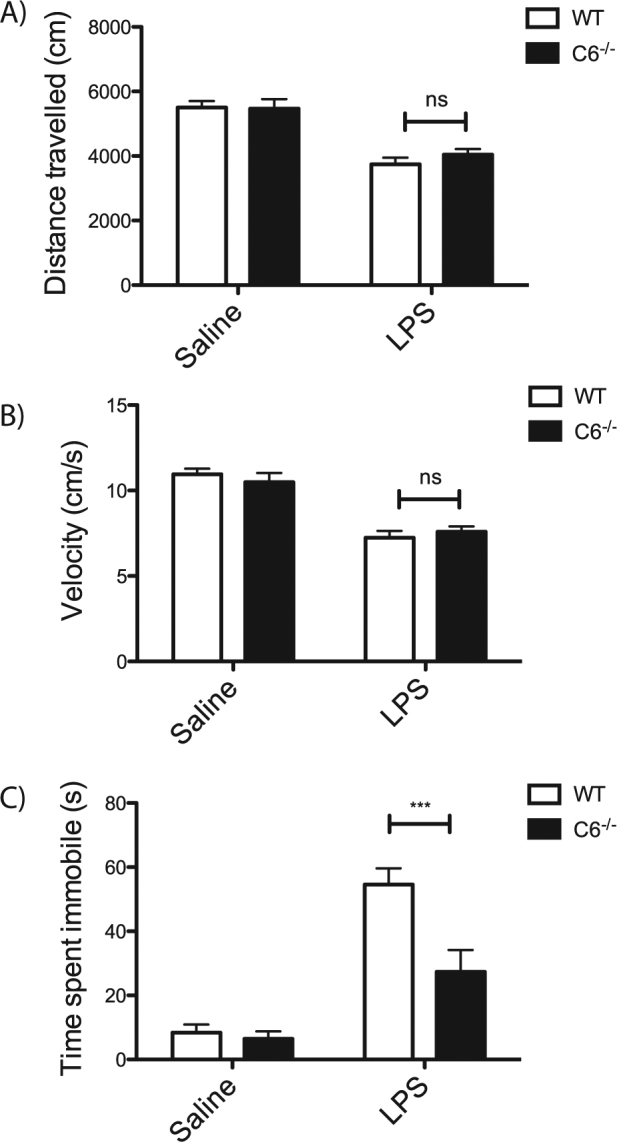
The loss of C6 attenuates LPS-induced depression in mice. Activity measurements of distance travelled (**a**, genotype *p* = 0.5571; treatment *p* < 0.0001; interaction *p* = 0.4733; *N* = 10) or velocity (**b**, genotype *p* = 0.8854; treatment *p* < 0.0001; interaction *p* = 0.3300; *N* = 10) using the open field test in WT and *C6*^*−/−*^ mice following treatment with saline or LPS demonstrate that no genotypic differences in activity exist following LPS treatment. Forced swim testing reveals that loss of C6 results in an attenuation of LPS-induced behavioural despair (**c**, genotype *p* = 0.0032; treatment *p* < 0.0001; interaction p = 0.0096; *N* = 10). Statistical significance was determined using a two-way ANOVA with post-hoc Bonferroni tests. *p < 0.05, **p < 0.01, ***p < 0.001

**Fig. 4:**
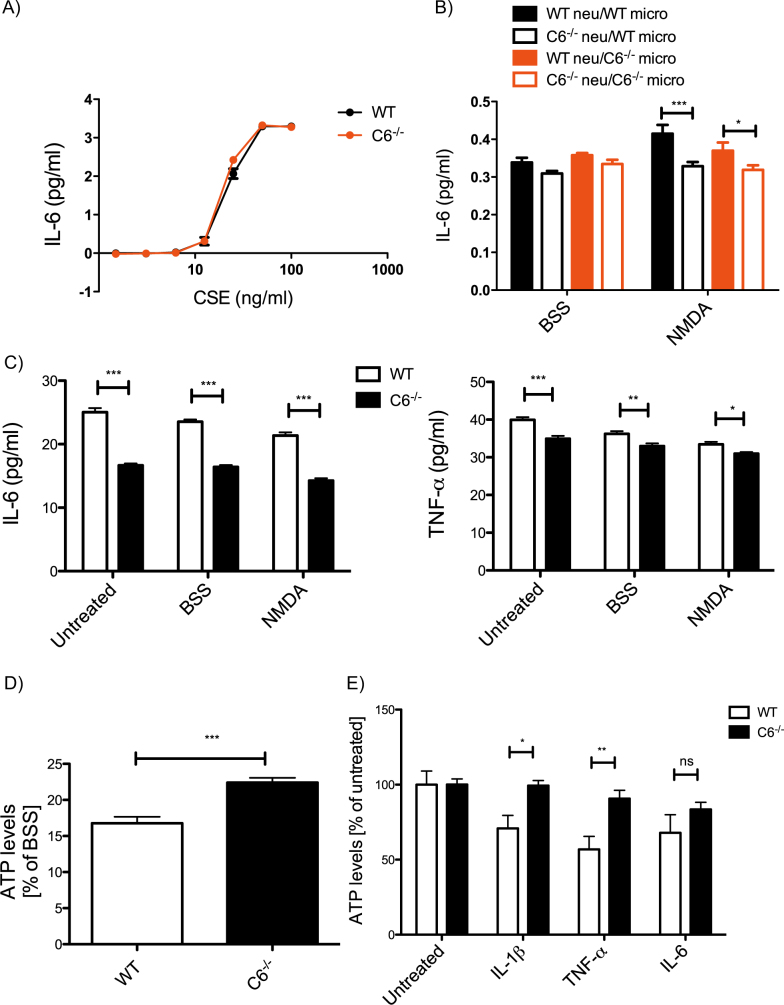
Neuronal C6 influences cytokine release and microglial inflammatory response. Microglia cultured from WT and *C6*^*−/−*^ mice do not display significant differences in IL-6 secretion following CSE stimulation (**a**, genotype *p* = 0.08, treatment *p* < 0.0001). Microglia stimulated with medium from NMDA-treated *C6*^*−/−*^ neurons demonstrate significantly reduced IL-6 secretion (**b**, genotype *p* = 0.0021; treatment *p* = 0.0505; interaction *p* = 0.0390). Untreated *C6*^−^^*/−*^ neurons as well as *C6*^*−/−*^ neurons treated with BSS or NMDA release less TNF-α and IL-6 than WT, irrespective of treatment (**c**, IL-6: genotype *p* < 0.0001; treatment *p* < 0.0001; interaction *p* = 0.2402; TNF-α: genotype *p* < 0.0001; treatment *p* < 0.0001; interaction *p* = 0.1720). ATP assays demonstrate that *C6*^−^^*/−*^ neurons are less impacted by NMDA treatment than WT neurons (**d**, *p* = 0.0002). ATP levels from WT and *C6*^*−/−*^ neurons following treatment with cytokines show that loss of C6 confers resistance to cytokine-induced toxicity (**e**, genotype *p* = 0.0004; treatment *p* = 0.0022; interaction *p* = 0.1071). Statistical significance was determined using a two-way ANOVA with post-hoc Bonferroni tests (**a, b, c, e**) or Student’s *t*test (**d**) and *N* = 3 independent cultures. *p < 0.05, **p < 0.01, ***p < 0.001. Asterisks refer to significant differences following *t*test or post-hoc analysis

We therefore injected WT and *C6*^*−/−*^ mice with LPS and subjected them to behavioural analyses. The mice were first tested in an open field test to measure overall activity in order to ensure LPS-induced sickness was not a confounding factor in behavioural performance. While LPS treatment did induce a slight reduction in both distance travelled (Fig. 3a) and velocity (Fig. 3b) in the open field, both WT and *C6*^*−/−*^ mice experienced this reduction to the same extent, confirming that there are no genotypic differences in locomotor activity following treatment with LPS. Next, the forced swim test was conducted on the mice to assess behavioural changes. Consistent with previous reports^23,34,38^, WT mice treated with LPS spent significantly more time immobile compared to saline-treated control mice. However, LPS-treated *C6*^*−/−*^ mice spent significantly less time immobile than the LPS-treated WT mice (Fig. 4c), demonstrating that the absence of C6 attenuates endotoxin-induced acute depressive-like behaviour in mice.

### Neuronal C6 influences cytokine release and microglial inflammatory response

Neuroinflammation can originate both from peripheral and central causes, since persistently elevated levels of cytokines due to peripheral inflammatory stimulation can exert CNS toxicity^35,39^. To determine if the differential response to inflammatory stimuli observed in *C6*^*−/−*^ macrophages extended to the resident immune cells of the brain, we examined IL-6 levels released by microglia upon stimulation with increasing doses of the inflammatory control standard endotoxin (CSE). Interestingly, IL-6 levels measured in the media following stimulation reveal no differences in the secretion of this cytokine between WT and *C6*^*−/−*^ microglia (Fig. 4a), suggesting the absence of C6 expression does not influence microglial-driven IL-6 activation or production in response to bacterial toxins.

In the CNS, neuronal damage is another relevant stimulus that induces microglial activation, in particular in the context of acute and chronic neurological disease. To simulate this scenario in vitro, cortical neurons from WT and *C6*^*−/−*^ mice were cultured and treated with either NMDA or balanced salt solution (BSS) as a control^40^. NMDA was chosen because it causes neuronal dysfunction and damage, which subsequently activates microglia^41^. The neuronal conditioned media was then added to the media of microglia cultured from WT and *C6*^*−/−*^ mice, combining media from WT neurons with *C6*^*−/−*^ microglia and vice versa in all four possible permutations. Media was harvested again the following day and analysed for IL-6 cytokine levels. Media from *C6*^*−/−*^ neurons did not elicit IL-6 release from microglial cultures, irrespective of NMDA treatment or the presence of C6 in microglia (hollow bars, Fig. 4b). However, media derived from NMDA-stimulated WT neurons led to IL-6 release from WT as well as *C6*^*−/−*^ microglia (filled bars, Fig. 4b). Although we observed a trend towards a weaker effect on *C6*^*−/−*^ microglia (filled red compared to filled black bars, Fig. 4b), the presence of C6 in NMDA-stressed neurons had a bigger impact on IL-6 release than the presence of C6 in microglia.

This suggests that the transplanted media from NMDA-treated WT neurons may contain neuronal-derived factors that were not present in the NMDA-treated *C6*^*−/−*^ neuronal media, which ultimately contributed to the increased secretion of cytokines by microglia. Although microglia and astrocytes are the cell types that are predominantly responsible for cytokine production in the CNS, neurons have been shown to produce TNF-α, IL-6 as well as other cytokines^42,43^.

We therefore checked whether NMDA treatment induced cytokine release from neurons, since these would be present in the medium that we transplanted onto microglia, and could lead to paracrine activation of microglia. Interestingly, we found that WT neurons secrete higher levels of both IL-6 and TNF-α compared to cells derived from *C6*^*−/−*^ mice (Fig. 4c). However, treatment with NMDA did not further increase the release of pro-inflammatory cytokines, making these an unlikely candidate for the induction of microglial responses to neuronal injury.

However, we found that *C6*^−^^*/−*^ neurons were more resistant to NMDA treatment, in agreement with published data^40^, as they did not lose viability to the same degree as WT cultures (Fig. 4d). This loss of viability may therefore be associated with the release of DAMPs causing microglial activation. Furthermore, WT neurons were also more sensitive than *C6*^*−/−*^ neurons to treatment with pro-inflammatory cytokines (Fig. 4e).

Taken together, these findings suggest that neurons lacking C6 are more resistant to a wide range of stimuli, ranging from pro-inflammatory cytokines that may enter the CNS after a peripheral inflammatory stimulus to excitotoxic damage, which is a hallmark of many acute and chronic neurological conditions^44^. This resistance would thus reduce the amount of DAMPs, microglial activation and neuroinflammation in the absence of C6, slowing down a toxic cycle of neuronal damage and pro-inflammatory stimuli.

## Discussion

Caspases are multi-functional enzymes involved in a wide variety of cellular pathways besides programmed cell death^6,9,10,45–50^. Recent evidence suggests that the executioner caspase C6 can regulate the production of pro-inflammatory cytokines after different stimuli^11,15^, which adds to the growing list of non-apoptotic functions for this enzyme. Here we report reduced levels of TNF-α in the plasma of *C6*^*−/−*^ mice, both at baseline and after stimulation with LPS. These effects are likely due to decreased expression of TNF-α in *C6*^−^^*/*^^−^ macrophages.

The regulatory effect of C6 furthermore seems to be specific to TNF-α, as the ablation of C6 had a greater effect on the transcription and release of TNF-α in the plasma following LPS stimulation. Differential mechanisms for TNF-α and IL-6 production have been proposed^51–57^. While LPS-induced synthesis of both IL-6 and TNF-α occurs via p38 activation and subsequent NFκB-mediated gene transcription, TNF-α is also produced following the generation of reactive oxygen species by a p38-independent mechanism^51^. Since we do not find changes in peripheral IL-6 levels in macrophages or plasma of *C6*^−^^*/−*^ mice, it is likely that such p38-independent transcriptional regulation is responsible for the specific reduction of TNF-α observed in this setting.

The attenuation of behavioural despair in mice lacking C6 suggests that C6 may be involved in the pathophysiology of affective behaviours, possibly through the modulation of inflammatory cytokines. Neuroinflammation, depression and the aberrant activation of C6 are hallmarks of Huntington disease (HD), a progressive neurodegenerative disorder caused by a mutation in the huntingtin gene. In this context we recently demonstrated that the depressive-like phenotype in mouse models of HD can be rescued by the ablation of C6^58^ or through the administration of a peptide inhibitor of C6^59^. Together with the findings of the current study, it is thus tempting to speculate that the depressive-like phenotypes in HD mouse models may be influenced by neuroinflammation due to aberrant C6 activity.

C6 activation can result from neuronal stress-induced NLRP1 inflammasome formation and activation of C1, which results in coordinated IL-1β-mediated neuroinflammation and C6-mediated axonal degeneration^13^. While the consequence of C6 activation in neurons is axonal degeneration^13^, it is possible that this process would generate DAMPs that trigger neuroinflammatory responses in glia. Intriguingly, links have also been suggested connecting excitotoxic and inflammatory pathways. These two pathways have been thought to converge upon TNF-α as TNF-α has been shown to potentiate glutamate-induced toxicity^60^ while also mediating inflammation following its release from microglia^61^. C6 has a well-established role in facilitating excitotoxicity as *C6*^*−/−*^ mice are protected from excitotoxicity and myelin-induced axonal degeneration^40^. Given that excitotoxicity can lead to NMDA receptor activation on microglia and downstream inflammatory signalling^41^, it is quite possible that C6 plays a previously underappreciated role in mediating inflammation. Specifically, we show data to support the hypothesis that the dampened inflammatory response in *C6*^*−/−*^ mice may not due to cell-autonomous differences in microglial cytokine release but potentially rather a paracrine response to reduced neuronal damage.

The overproduction of cytokines including TNF-α basally or following stimulation in the periphery can have consequences on CNS function and neuronal health. Cytokines can cross the BBB, and the exposure of neurons to individual or a combination of cytokines leads to lactate dehydrogenase release, reduced adenosine triphosphate (ATP) and ultimately nitric oxide-mediated apoptosis^62,63^. We show that the ablation of C6 in neurons confers resistance to cytokine-induced toxicity, a protective mechanism that promotes survival of neurons in the face of inflammation. Thus, it would seem that the loss of C6 confers dual protection against inflammatory insults in *C6*^*−/−*^ mice, first at the peripheral level in the attenuation of TNF-α production and subsequently at the neuronal level in the protection against cytokine-induced apoptosis.

## Materials and methods

### Animals

Caspase-6 constitutive knockout mice (*C6*^−^^*/−*^) on the FVB background were previously described^40^. *C6*^−^^*/−*^ mice were genotyped using the following primers: 5′-CCTGTGGGGTCAAAAGACTTTCACAG-3′ (forward) and 5′-GCAAGCTGCTAACAGCCAACACAAC-3′ (reverse). Due to reported sex differences in neuropsychiatric phenotypes including depression in rodents^64,65^, only female animals were included in this study with the exception of pups used for primary cultures, which were of both sexes. All animal experiments were conducted according to protocols approved by the Animal Care Committee at the University of British Columbia (protocol number A16-0130).

### LPS injections, plasma collection and cytokine ELISAs

Lipopolysaccharide (LPS) derived from *Escherichia coli* (Sigma L2630 serotype O111:B4) was dissolved in 0.9% sterile saline. Two to 3-month-old wild-type (WT) and *C6* knockout (*C6*^−^^*/−*^) mice were intraperitoneally injected with 0.5 mg/kg LPS, a dose that effectively induces inflammation without causing behaviour-impairing sickness, consistent with previous studies^34,66,67,]^. For plasma isolation, blood was collected from the saphenous vein just before LPS treatment as well as 1 h post-LPS injection in EDTA-coated tubes (Sarstedt, Numbrecht), kept on ice and centrifuged at 6000 rpm and 4 °C for 10 min. Clear supernatant was then removed for analysis. Cytokine enzyme-linked immunosorbent assays (ELISAs) for IL-6 and TNF-α were conducted according to the manufacturer’s instructions (eBioscience, 88-7064-86 and 88-7324-88, respectively).

### Behavioural analyses

All behaviour-tested mice were housed on a 12 h light/dark cycle and the experimenter was blind to all genotypes. All behaviour analyses were conducted during the dark phase of the cycle. Behaviour-tested mice were injected with 0.5 mg/kg LPS and tested for activity and depression-like behaviour 24 h later. Activity was assessed using the open field as previously described^68^ and consisted of placing the mice in an empty 50 × 50 cm black Plexiglas box with 16 cm high edges in a brightly lit room. Mice were allowed to explore the box and were tracked using EthoVision^®^ XT 7 tracking software (Noldus). The total distance travelled and average velocity was recorded. Immediately following assessment of activity, mice were tested for depression using the Porsolt forced swim test as previously described^69,70^. Mice were placed in transparent cylinders 25 cm tall × 19 cm wide filled with room temperature water to a depth of 15 cm. Mice were videotaped for 6 min using a camera located above the cylinders, with the last 5 min scored for time spent immobile as a readout for behavioural changes.

### Peritoneal macrophage harvest and culture

Peritoneal macrophages were elicited with Brewer’s thioglycollate treatment as previously described^26^. Mice were intraperitoneally injected with 3% Brewer’s thioglycollate (BD, 211716) and left in their home cages for 5 days to allow for macrophage migration to the peritoneal cavity. Mice were then killed by CO_2_ asphyxiation and the abdominal skin retracted to expose the intact peritoneal wall. Approximately 5 ml of ice-cold PBS was injected into the peritoneal cavity, after which the peritoneum was gently messaged to disperse liquid and the fluid was aspirated back from the peritoneum slowly. This lavage was repeated 2–3 times and the extracted fluid was centrifuged at 1200 rpm and 4 °C for 10 min. The cell pellet was re-suspended in warm RPMI 1640 media (Invitrogen) containing 5% FBS, 1% penicillin/streptomycin and plated at a density of approximately 1×10^6^ cells/well in a six-well plate. Cells were incubated at 37 °C, 5% CO_2_ and media was replaced 2–3 h later to remove non-adherent cells. Following 24 h in culture, peritoneal macrophages were harvested and subjected to quantitative RT-PCR.

### Quantitative real-time PCR

Total RNA was extracted from peritoneal macrophage pellets using the RNeasy micro kit (Qiagen, 74004). Residual genomic DNA was destroyed using DNAse I (Invitrogen, 18047-019) and cDNA was prepared from 250 ng to 1 μg total RNA using the SuperScript^®^-III First-Strand Synthesis kit (Life Technologies, 18080-051). Quantitative real-time PCR was performed using the Power SYBR Green PCR master mix (Applied Biosystems, 4309155) in the ABI 7500 Fast instrument (Applied Biosystems) under default conditions in triplicate. The primers used are listed in Table 1.

**Table 1:** List of primers used in quantitative real-time PCR

Gene	Forward primer (5′-3′)	Reverse primer (5′-3′)
*C6*	TTTAACGACCTCAGAGCAGAAG	GGCTCAGGAAGACACAGATG
*Rpl13a*	GGAGGAGAAACGGAAGGAAAAG	CCGTAACCTCAAGATCTGCTTCTT
*IL-6*	TCGGAGGCTTAATTACACATGTTCT	GCATCATCGTTGTTCATACAATCA
*TNF-α*	ATGAGAAGTTCCCAAATGGCC	TCCACTTGGTGGTTCGCTACG

### Neuronal culture and treatment

Embryonic day 15.5–17.5 cortical neurons were cultured as previously described^71^ from timed-pregnant FVB and *C6*^*−/−*^ females. Cultures were incubated at 37 °C and 5% CO_2_ and half the media was changed every 4–5 days. On the tenth day in vitro, cells were treated with 5 ng/ml IL-1β or TNF-α, or 2 ng/ml IL-6 for 24 h. The media was removed and neurons were subjected to an ATP assay (Cell Titer Glo, Promega) carried out according to the manufacturer’s instructions. For NMDA treatment, medium was removed from neurons at day 10 in vitro and cultures were treated with 500 μm NMDA in BSS (138 mm NaCl, 3.5 mm KCl, 1.8 mm CaCl_2_, 11 mm d-glucose, 10 mm HEPES pH 7.4, 3 mm Na_2_HPO_4_, 50 μm glycine, 2 mm NaHCO_3_) or BSS only as a control for 10 min. Then, the original medium was added back to each well and cells were incubated for further 48 h. Conditioned medium was stored at −80 °C until it was added to microglial cultures or analysed for cytokine content by ELISA as described above. Cell viability was determined using an ATP assay as described above.

### Microglial culture and treatment

Microglia were isolated from individual hemispheres of 0- to 2-day-old post-natal mice. Hemispheres were minced, triturated, centrifuged and plated in DMEM media (Invitrogen). Ten to 14 days later, the mixed cultures were gently shaken for 4 h and floating cells (microglia) were collected and plated separately in DMEM. Cells were seeded into a 96-well plate and the following day switched to media containing 1% FBS. The next day, microglia were treated with varying concentrations of CSE (Associates of Cape Cod) combined with 2.5 ng/μl interferon-gamma (IFN-γ) for 24 h. Following treatment, the media was harvested for the quantification of cytokines. For treatment with conditioned neuronal media, microglia were incubated for 1 day in 1% FBS-containing media (100 μl/well) before 50 μl/well neuronal media was added. Mixed media was harvested 24 h after treatment for the quantification of cytokines.

### Statistical analysis

Data were analysed using Prism 5 statistical software and error bars represent standard error of the mean. For data with one independent variable, a Student’s unpaired *t*test was conducted. For two independent variables, a two-way ANOVA was used. Bonferroni’s post-hoc tests were conducted following 1- or 2-way ANOVA analyses. Asterisks refer to significant differences by *t*test or by post-hoc following an ANOVA (as appropriate) unless otherwise indicated.
